# Spectral Measurements of Muzzle Flash with a Temporally and Spatially Modulated LWIR-Imaging Fourier Transform Spectrometer

**DOI:** 10.3390/s23083862

**Published:** 2023-04-10

**Authors:** Zhixiong Yang, Kun Li, Chunchao Yu, Mingyao Yuan, Boyang Wang, Jie Feng

**Affiliations:** 1 College of Physics and Electronic Information, Yunnan Normal University, Kunming 650000, China; 2 Kunming Institute of Physics, Kunming 650223, China

**Keywords:** gas detection, fourier transform imaging spectrometer, hyperspectral

## Abstract

It is important to obtain information on an instantaneous target. A high-speed camera can capture a picture of an immediate scene, but spectral information about the object cannot be retrieved. Spectrographic analysis is a key tool for identifying chemicals. Detecting dangerous gas quickly can help ensure personal safety. In this paper, a temporally and spatially modulated long-wave infrared (LWIR)-imaging Fourier transform spectrometer was used to realize hyperspectral imaging. The spectral range was 700~1450 cm^−1^ (7~14.5 μm). The frame rate of infrared imaging was 200 Hz. The muzzle-flash area of guns with calibers of 5.56 mm, 7.62 mm, and 14.5 mm were detected. LWIR images of muzzle flash were obtained. Spectral information on muzzle flash was obtained using instantaneous interferograms. The main peak of the spectrum of the muzzle flash appeared at 970 cm^−1^ (10.31 μm). Two secondary peaks near 930 cm^−1^ (10.75 μm) and 1030 cm^−1^ (9.71 μm) were observed. Radiance and brightness temperature were also measured. The spatiotemporal modulation of the LWIR-imaging Fourier transform spectrometer provides a new method for rapid spectral detection. The high-speed identification of hazardous gas leakage can ensure personal safety.

## 1. Introduction

The measurement of a radiating scene’s spectrum is a fundamental diagnostic tool in the physical sciences, with applications in physics, chemistry, medicine, and remote sensing [1,2,3,4]. The measured spectrum enables the sensing of the properties of the scene and is the main source of information regarding the chemical composition and physical properties of the targeted objects. The leakage of dangerous gas seriously threatens the safety of people and the environment. The detection of toxic and harmful gases requires an ultrafast response speed. Therefore, an ultrafast gas detection system is of great significance for environmental protection and personal safety. Research on instantaneous scenes such as explosions, combustion, and trajectories has important applications in the field of national defense science and technology [5,6]. The phenomena detected in infrared spectra are muzzle flashes and the thermal signatures of bullets in flight [7]. The object of detecting the signature of a bullet in flight is to estimate the bullet’s trajectory and backtrack its path to determine the location of the shooter. The spectral radiance of the flash, the spectral content of the gun powder, and the spectral shapes/geometries of the muzzle flashes are analyzed. After a more complete database is formed, it is possible to develop a passive electro-optical weapon and ammunition identifier [8]. At the moment of firing, the temperature signature and spectrum of the muzzle flash of light weapons exist for a short time. The sound wave of a gunshot has a great impact via strong vibrations on the surrounding objects. Therefore, it is difficult to measure the temperature and spectrum of muzzle flash accurately. A high-speed infrared sensor has been developed by Andrew Ashcroft et al. [9]. The sensor offers >1000 fps in a full 384 × 384 format and useful images at up to 6500 fps in smaller formats. A mid-wave infrared image of the muzzle of the rifle was obtained using the sensor. M. Kastek et al. obtained muzzle flash images at 3–5 μm and analyzed the temperature change during the shot with seven different measurement devices [10]. At present, the main method of image spectrum acquisition is computational optics, which has a long imaging period and poses difficulty with respect to obtaining a fast and instantaneous spectrum. In particular, due to the limitations of technical means and weak signals, the fast and instantaneous long-wave infrared spectrum at room temperature was difficult to obtain.

There are three main approaches to infrared interference imaging: temporally modulated, spatially modulated, and temporally and spatially modulated. The products of temporally modulated Fourier transform infrared spectroscopy technology are relatively mature. Bruker’s HI90 [11] and Telops’s HyperCAM [12] are applied in chemical gas sensing. Due to the limitation of the detector, the development of longwave infrared spectroscopy is relatively slow. A spatially modulated Fourier transform infrared-imaging spectrometer produces interference fringes on the focal plane of the detector. The interference does not depend on the relative position of the moving mirror. The temporally and spatially modulated-imaging Fourier transform spectrometer has no moving mirrors or slits; thus, it has the advantages of multi-channel, high-luminous-flux, and static interference properties. The Kunming Institute of Physics has made progress with respect to research into the temporally and spatially modulated-imaging Fourier transform spectrometer [13]. It has been applied in the chemical remote sensing of gasses such as ether, ammonia, SF_6_, and DMMP [14].

In this paper, a temporally and spatially modulated long-wave infrared (LWIR)-imaging Fourier transform spectrometer was used to obtain the infrared spectrum of light weapons when shooting. The main peak of the spectrum of the muzzle flash appeared at 970 cm^−1^ (10.31 μm), and there were also two secondary peaks near 930 cm^−1^ (10.75 μm) and 1030 cm^−1^ (9.71 μm). Radiance and brightness temperature values were also obtained.

## 2. The Experimental System

**Figure 1:**
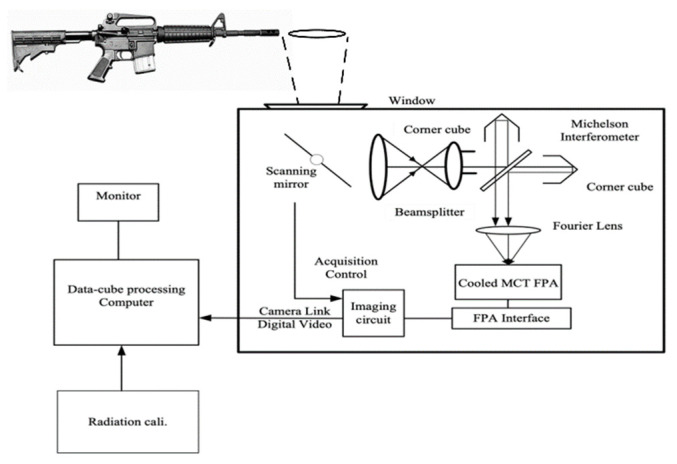
The experimental system for measuring the spectrum of muzzle flash.

The layout of the experimental system for measuring the spectrum of muzzle flash is shown in Figure 1. A temporally and spatially modulated LWIR-imaging Fourier transform spectrometer is used. It consists of a scanning mirror, an initial imaging system, a beam splitter system, two corner cubes, a second imaging system, and an LWIR detector. A 320 × 256 cooled mercury cadmium telluride focal plane array was used. The incident scene radiation forms interference fringes through the Michelson interferometer, which are superimposed on the infrared image. There is an optical path difference between the two beams of light incident on the detector, so the image received by the detector contains both spatial information and spectral information on the object. The scanning mirror is swung according to the interval of an instantaneous field angle of view (IFOV) in the spectral dimension. Each column of pixels in the spectral dimension needs to collect the interference information from the scene, so 640 infrared image sequences with equal time intervals need to be collected to form the original window-scanning image data cube. Then, the interference cube data’s reorganization, correction, FFT transformation, etc., are carried out to obtain a spectral data cube [15].

For the resolution of a 320 × 256 target, the detector collects 640 interferograms in succession, moving from the left side of the field of view to the right side. According to the 200 Hz sampling rate of the focal plane detector, this process takes 3.2 s. Each image frame is composed of an infrared image of the target and its corresponding interference fringe. The obtained multi-image frames form an infrared interference data cube. Since it is both an interference image and a scene image, the cube data are referred to as an image interference cube.

**Figure 2:**
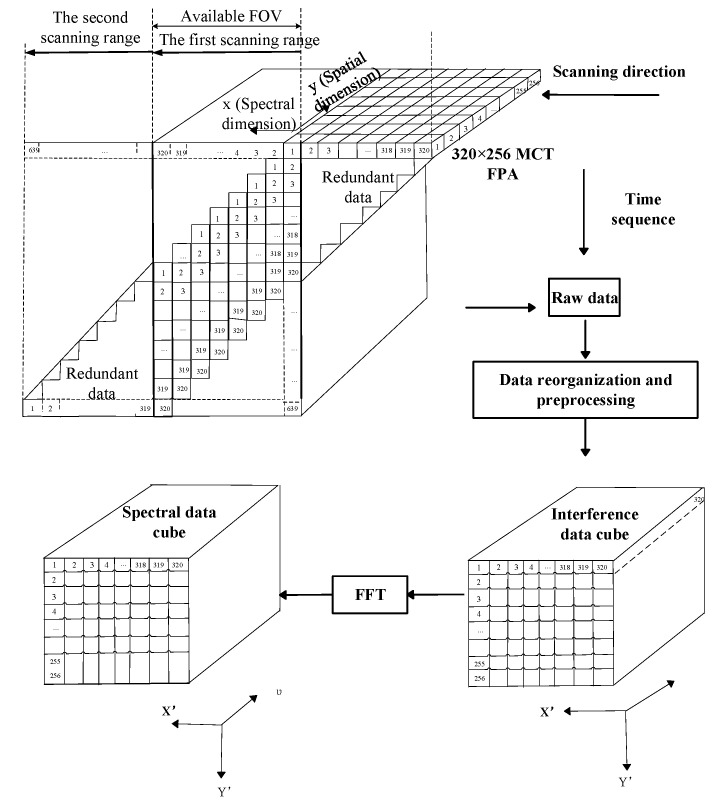
The Schematic of TSMFTIS data processing.

A schematic of temporally and spatially modulated-imaging Fourier transform spectrometer’s (TSMFTIS) data-processing scheme is shown in Figure 2. A spectral image data cube can be obtained by using a one-dimensional FFT transform for each row in each frame of the interference image cube data. If the original interference frame data form a single-sided interferogram, to carry out FFT transformation, the single-sided data must be symmetrically processed. The interferogram data, after being symmetrically processed, are a double-sided distribution sequence, and the mode of Fourier transform represents the spectral distribution to be measured [16]:

(1) $$F\left[I\left(l\right)\right]=\sum_{l=0}^{N-1}f\left(l\right)\exp\left(-j\frac{2\pi lk}{N}\right),k=0,1,2,3\ldots N-1$$

where *ν* is the wave number, *l* is the pixel spacing, and *N* is the order of the interferogram.

To obtain spectral images, the interference data cube is processed by interference date filtering and toe cutting, and then the spectral data cube images can be obtained by a complex Fourier transform. At this time, the obtained spectral information is only relative spectral information, and the wavelength information of each band can only be obtained after spectral calibration.

**Figure 3:**
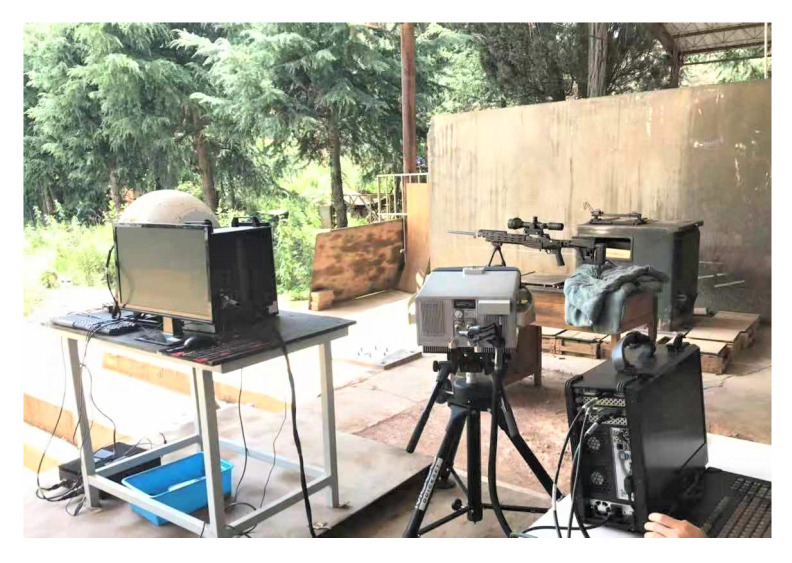
Physical diagram of the experimental system.

The physical diagram of the experimental system is shown in Figure 3. The spectral range was 700~1450 cm^−1^ (7~14.5 μm) of the TSMFTIS. The frame rate of infrared imaging was 200 Hz, so the time interval of the long-wave infrared acquisition was 5 ms. Considering the influence of the sound waves when shooting and the uncertainty at the beginning of image acquisition, three experiments were carried out for each caliber weapon. One clear interferogram was selected from the playback data, and 640 frames of the still image were filled as the original interferogram. The original data cube was reconstituted and FFT processing was carried out to obtain the spectral data cube. Before the experiment, the standard sample film and the extended area blackbody were used for wavelength correction.

## 3. Results and Discussions

### 3.1. The Interference Diagram of Muzzle Flash

**Figure 4:**
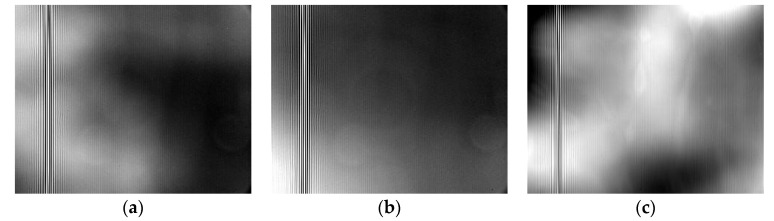
Interferograms of muzzle flash when firing. (**a**) 5.56 mm caliber; (**b**) 7.62 mm caliber; (**c**) 14.5 mm caliber.

Figure 4 shows the interferogram of the muzzle flash of the light weapons at the moment of firing. Due to the impact of the sound wave when firing, some of the obtained images were invalid. Consequently, we only selected some interferograms. Therefore, the FFT processed spectrograms only show spectral dimension information, while spatial dimension information is missing. In the test, a time-modulated spectrometer (HYERCAM-LW) was used for a comparative experiment. Spectral data could not be acquired when firing. After the firing of 14.5 mm sniper rifle, there was a short-term failure, which was recovered after a restart. The sound volume was high on site, which might have been caused by large shockwaves and the vibration of the sound waves. The interference fringes formed by time modulation depend on the time series of the moving mirror positions, so anti-vibration capacity was relatively poor.

### 3.2. The Spectra of Muzzle Flash

**Figure 5:**
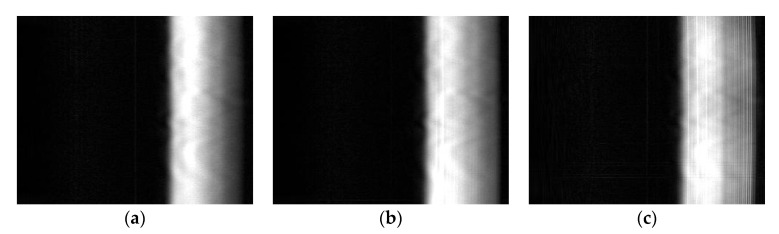
Long-wave infrared radiation spectrum of muzzle flash when firing. (**a**) 5.56 mm caliber; (**b**) 7.62 mm caliber; (**c**) 14.5 mm caliber.

After the Fourier transform of the selected interferogram, the long-wave infrared radiation spectrum of the muzzle flash of the light weapon at the moment of firing was obtained, as shown in Figure 5. The horizontal direction of the image represents the wavelength, and the gray value of the image represents the spectral intensity. Since the working wavelength of the detector was long-wave infrared, the intensity of the spectrum at the short-wave range is zero. The short-wave range is shown in black, and the long-wave infrared range is depicted in white.

**Figure 6:**
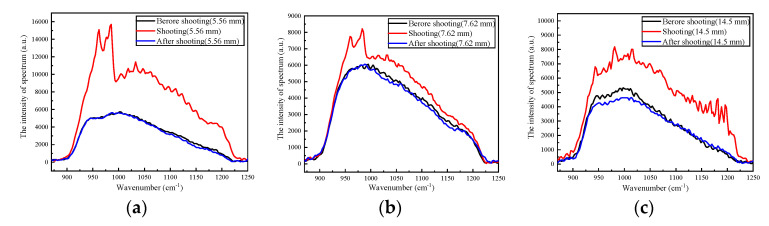
The intensity of the spectrum of muzzle flash. (**a**) 5.56 mm caliber; (**b**) 7.62 mm caliber; (**c**) 14.5 mm caliber.

The intensity of the spectrum of the muzzle flash is shown in Figure 6. For the three different caliber weapons, the intensities of the spectra before and after shooting were almost the same. It could be regarded as the background spectrum. The peak of the background spectrum was around 1030 cm^−1^. The peaks of the spectra of the muzzle flash of the three kinds of light weapons when shooting were around 970 cm^−1^ (10.3 μm). There were two secondary peaks near 930 cm^−1^ (10.75 μm) and 1030 cm^−1^ (9.71 μm). The peak of each spectrum has a certain relationship with the composition of the powder. The main component of gunpowder is potassium nitrate (KNO_3_). According to the NIST spectral database, KNO_3_ is responsive in the 700 cm^−1^~1450 cm^−1^.

### 3.3. The Radiance of Muzzle Flash

**Figure 7:**
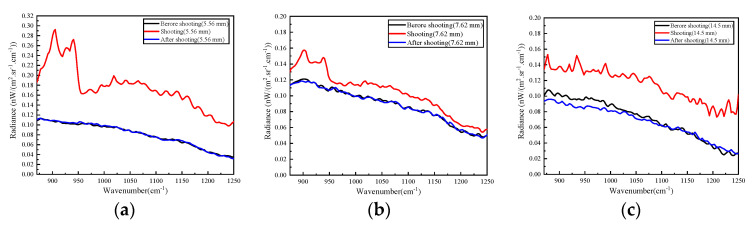
The radiance of muzzle flash. (**a**) 5.56 mm caliber; (**b**) 7.62 mm caliber; (**c**) 14.5 mm caliber.

The radiance of the muzzle flashes is shown in Figure 7. Although the observation position of the instrument was adjusted before each gunshot, the sound waves also caused certain vibrations. The spectral intensity of the 5.56 mm caliber light weapon’s muzzle flash was the strongest. It might be that the selected snapshot’s instantaneous field of view was in just the right muzzle observation position. There were several maximum spectral peaks near 1180~1200 cm^−1^ in the short-wave direction in the spectrum of the 14.5 mm caliber light weapon’s muzzle flash, which indicated that the temperature was high.

### 3.4. The Brightness Temperature of Muzzle Flash

**Figure 8:**
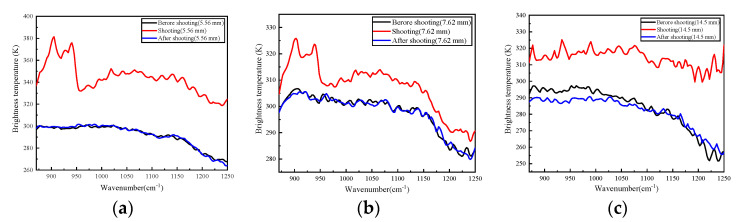
The brightness temperature of muzzle flashes. (**a**) 5.56 mm caliber; (**b**) 7.62 mm caliber; (**c**) 14.5 mm caliber.

The brightness temperature was of great significance in the remote-sensing experiment. After correction by a blackbody, the brightness temperatures of the radiation of the spectra were obtained, as shown in Figure 8. The instantaneous radiation brightness temperature of the 5.56 mm caliber gun was the highest. It might be that the selected interference picture was taken at the moment of the weapon’s discharge. The instantaneous radiation brightness temperature of the 14.5 mm caliber gun was the same in the longwave infrared region. It might be that the larger caliber gun’s discharge involved a higher degree of energy. When it was firing, the temperature of the muzzle airflow was high.

### 3.5. Gas Telemetering

**Figure 9:**
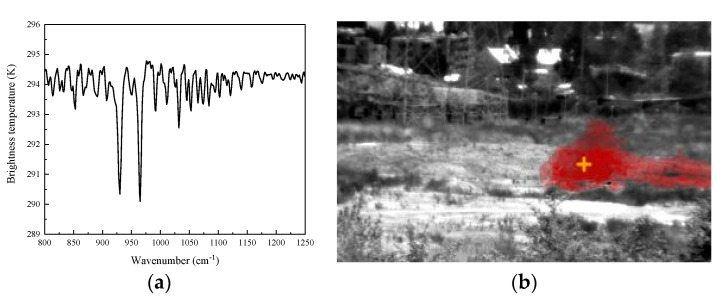
Remote measurement of ammonia. (**a**) The brightness temperature of ammonia; (**b**) identification results regarding ammonia. Explanation of +: The crosshair is the predicted leakage point.

The imaging spectrometer was used to detect the ammonia released at a distance of 1 km. As shown in Figure 9a, two absorption peaks of ammonia can be seen, which are consistent with the standard absorption spectrum of ammonia. As shown in Figure 9b, the spatial distribution and diffusion trend of ammonia can be clearly identified. The imaging spectrometer provided a remarkable method for the detection of toxic and harmful gases.

## 4. Conclusions

A Fourier-imaging spectrometer with a high sampling rate can acquire spectral information of instantaneous targets. Accordingly, the leakage of dangerous gas can be promptly detected, and this information can be used to trigger an alarm in order to protect the safety of personnel. Spectral measurements of muzzle flash are of great significance in ultrafast gas detection. Through the snapshot of a temporally and spatially modulated-imaging Fourier transform spectrometer, the selection of a suitable interferogram, and the analysis of the spectral data, the instantaneous spectral change of light weapons can be ascertained. This method provides a possible means for the measurement of long-wave spectral images though a fast process. The technology of temporally and spatially modulated interference spectroscopy offers unique advantages in a high-vibration environment. Since only three shots were fired for each type of gun, there were only nine groups of data in total; thus, there were fewer observed data. The imaging frame rate of the detector was 200 Hz, so it could only reflect the spectra with an interval of 5 ms. Improving the frame rate of the imaging spectrometer could improve the capture of spectral images of transient processes.

## Data Availability

Not applicable.
